# Computational and experimental evaluation of the Tic-Tac-Toe RF coil for 7 Tesla MRI

**DOI:** 10.1371/journal.pone.0209663

**Published:** 2019-01-10

**Authors:** Narayanan Krishnamurthy, Tales Santini, Sossena Wood, Junghwan Kim, Tiejun Zhao, Howard J. Aizenstein, Tamer S. Ibrahim

**Affiliations:** 1 University of Pittsburgh, Department of Bioengineering, Pittsburgh, PA, United States of America; 2 Siemens Medical Solutions, New York, NY, United States of America; 3 University of Pittsburgh, Department of Psychiatry, Pittsburgh, PA, United States of America; 4 University of Pittsburgh, Department of Radiology, Pittsburgh, PA, United States of America; Universitat Duisburg-Essen, GERMANY

## Abstract

A variety of 7 Tesla RF coil systems have been proposed to produce spin excitation (B_1_^+^ field) and MR image acquisition. Different groups have attempted to mitigate the challenges at high and ultra-high field MRI by proposing novel hardware and software solutions to obtain uniformly high spin excitation at acceptable RF absorption levels. In this study, we extensively compare the designs of two distributed-circuit based RF coils: the Tic-Tac-Toe (TTT) head coil and TEM head coil on multiple anatomically detailed head models and in-vivo. Bench measurements of s-parameters and experimental B_1_^+^ field distribution were obtained in volunteers and compared with numerical simulations. RF absorption, quantified by both average and peak SAR, and B_1_^+^ field intensity and homogeneity, calculated/measured in terms of maximum over minimum and coefficient of variation (CV) in the region of interest (ROI), are presented for both coils. A study of the RF consistency of both coils across multiple head models for different RF excitation strategies is also presented.

## Introduction

Evaluating Ultra-high field (UHF) MR/MRSI at 7 tesla (T) and higher as a translational clinical tool has been the focus of several research groups [1–8]. The main advantages of UHF strengths are higher signal-to-noise ratio (SNR), higher spatial resolution, increased sensitivity to T1 & T2 contrast mechanisms and to magnetic susceptibility or blood oxygenation (BOLD) [9–12]. However, the main challenges with UHF strengths remain to be radiofrequency (RF) inhomogeneity and safety restrictions due to subject specific variation and increased power deposition or specific absorption rate (SAR) [13, 14].

At UHF frequencies, the human head size becomes comparable to the RF wavelength (at 7T, the wavelength is approximately 13cm in the brain tissues). As a result, the electromagnetic interactions between the human body/head and the RF coil become increasingly sensitive to variations in the size/shape of the sample [14]. Such interactions can lead to significant variations in the distribution/intensity of the circularly polarized component responsible for excitation (B_1_^+^) field as well as specific absorption rate (SAR) across different subjects. This issue in addition to the inherent electromagnetic field inhomogeneity and elevated RF power deposition associated with UHF human imaging can have detrimental effects on the quality and safety in high field MRI. Moreover, it is important to assure that the RF excitation does not result in localized SAR across different subjects [15]. While many electromagnetic simulation tools are currently used to calculate estimates of SAR for RF shimming purposes [16, 17], B_1_^+^ fields are typically experimentally measured/mapped in an experimental setting.

The adoption of multichannel RF coil designs for UHF MRI allows for multiple degrees of freedom in manipulating the RF fields [18]. As a result, several designs of RF transmit arrays have been proposed to improve RF coil performance, mainly evaluated in its capability to produce homogenous B_1_^+^ field distributions at acceptable levels of RF tissue absorption [19–21]. It is worth noting however, that several MR sequences and pulse designs can also improve the homogeneity of the spin excitation (as opposed to B_1_^+^ field distribution), such as adiabatic pulses [22, 23], tailored pulses [24, 25], and transmit SENSE [26, 27] or interleaved excitation of the modes with TIAMO [28, 29].

Many studies have been done to evaluate coil performance and safety assurance at 7T. Wolf et.al. 2013 carried out a comprehensive study of different human body models at 7T using a 16-element band-pass Birdcage coil, to assess how much detail is needed to accurately predict local SAR. In addition to looking at current distributions and SAR in different models, the paper compared local SAR hotspots in Duke (male) and Ella (female) virtual family models. Wang et.al. [30] compared B_1_^+^ homogeneity and SAR in a sphere at 7T for a 16-element birdcage, transverse electromagnetic resonator (TEM) and microstrip coils. The results showed that the B_1_^+^ homogeneity in the central axial plane of the sphere in the TEM coil was most homogeneous, with marginal difference in B_1_^+^ inhomogeneity between the microstrip and birdcage coils.

In this work, we study RF characteristics due to different human heads and compare different coils that are used at 7T. Specifically, the transverse electromagnetic resonator (TEM) coil [31] and 5-sided Tic-Tac-Toe (TTT) coil [32, 33] are evaluated via RF simulations using different anatomically detailed human head models and measurements utilizing a network analyzer and in-vivo 7T B_1_^+^ mapping on different human heads. We chose the 16-element TEM and TTT designs due to the availability of both coils as well as FDTD coil models in our facility and because they are based on distributed-circuit approach and have shown to produce the homogenous B_1_^+^ field distributions at 7T [34, 35]. The detailed analysis is performed through comparing finite-difference time-domain (FDTD) calculated and measured coil impedance, B_1_^+^ field (homogeneity and intensity) and SAR (average and local) across different heads for both coils.

## Materials and methods

### Coils

#### TTT coil design

**Fig 1:**
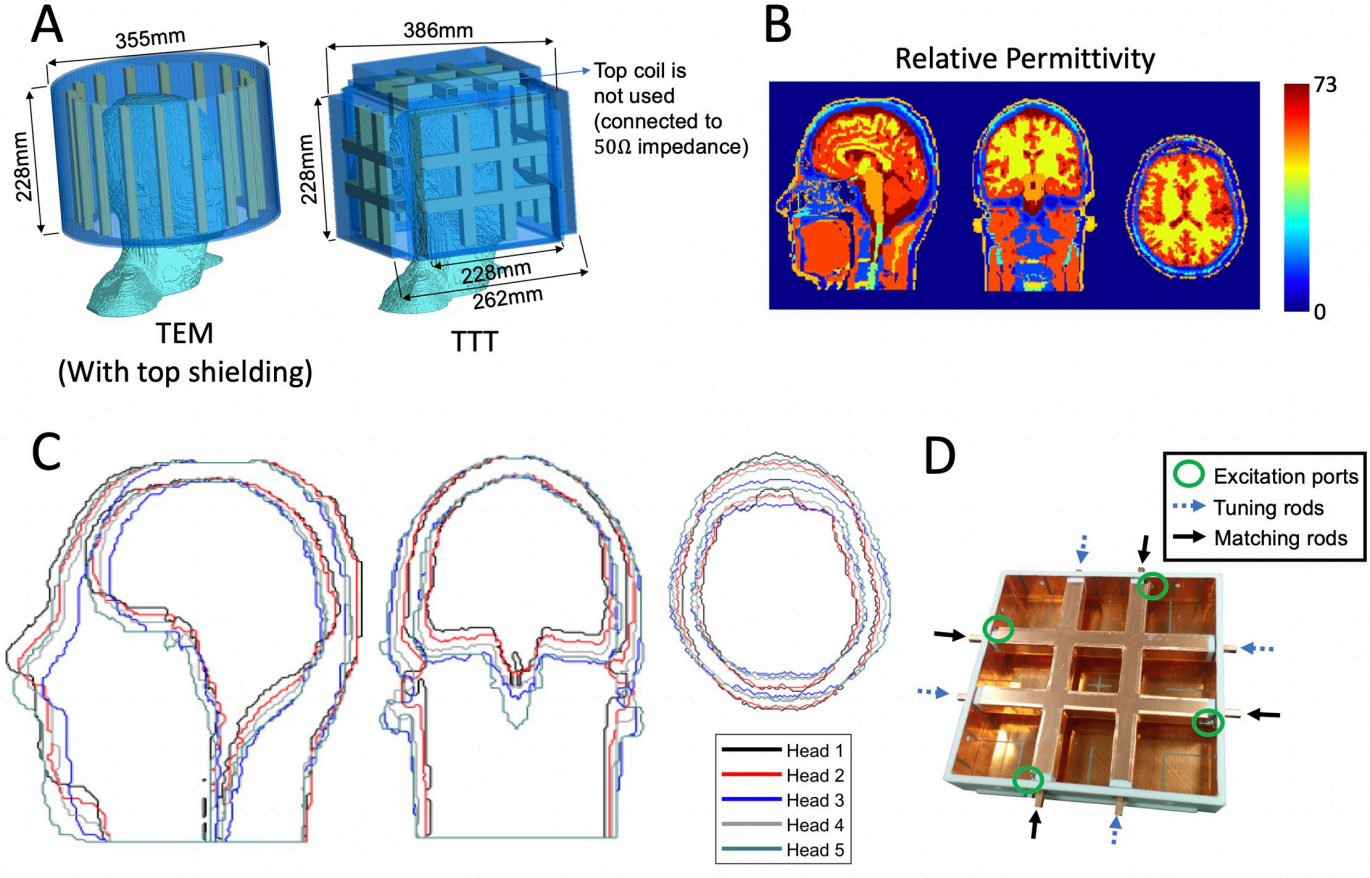
Coil designs and head models. In A, the TEM and TTT coil designs with anatomically detailed head model as the load; in B, the relative permittivity maps of one of the head models; In C, the superimposed outlines of all the head models; In D, a constructed TTT coil side.

Fig 1(A) shows the prototyped cuboid coil. The coil is made out of five sides of a TTT square-shaped array as described in [36–39]. The 7T TTT head coil is a bigger version (228x228 mm^2^) of the breast coil; detailed description of the coil design and construction can be found in [37]. One side of a tic tac toe shaped array has four transmit (Tx) channels/elements (Fig 1(D)). The assembly of 5 tic tac toe shaped arrays (surrounding the human head except for the neck side) allows for up to 20 Tx channels/elements. Only 16 Tx channels are used since the top tic tac toe side is not utilized. Each channel of the coil is tuned to 297.2 MHz by adjusting the inner rod length pushed inside the outer strut for all the coil elements.

#### TEM coil design

The TEM coil [31] consists of an array of transmission line elements (coaxial) with a cylindrical and end cap RF shielding. While, birdcage coils are popular with body imaging with larger ROI at 1.5T and 3T field intensities, TEM coils with 8, 16, and 24 transmission line elements have been built and tested for imaging the head at 7T [40]. It is worth noting that the commercial birdcage head coil (Nova Medical, Wilmington, MA, USA) is currently the most used RF coil for head imaging at 7T. Detailed analysis of a 16-element TEM design have shown good homogeneity in neuroimaging and its ability to tune over a broader frequency range, make it suitable for high field applications such as 7T. The four coaxial Tx ports of the 16-element TEM coil are tuned to 297.2 MHz by adjusting the inner rod length pushed inside the outer strut for all the coil elements. This coil, like 7T TTT design described earlier, is 228 mm long, it has an outer diameter of 355 mm and an inner diameter of 279 mm.

#### Coil construction

Both the TTT and the TEM Tx coils use double-layered copper sheets (each layer is 4um 38.1 gram/m^2^ Cu thickness with 0.254 mm dielectric between the layers.) For the TTT coil, the tic tac toe square-shaped array is made from (19.05 mm)^2^ Polycarbonate dielectric with (6.35 mm)^2^ inner opening for the inner rods. For the TEM coil, the elements are made of cylindrical-shaped (diameter = 12.7 mm) Teflon dielectric with inner opening (diameter = 6.35 mm) for the inner rods. The TEM elements are concentric with 279 mm diameter.

The excitation channels of each coil are combined using Wilkinson power dividers (2-, 4-, and 8- ways), with phase cables to implement particular RF shim phases (more on that in the “Excitation Strategy” section).

### RF modeling

#### Anatomically detailed human head models

Five anatomically detailed head models varying in shape/volume and weight were used to analyze the transmit coil characteristics of the TEM and TTT transmit coils at 7T using FDTD simulations. The anatomically detailed head models H1-5 were created from Duke male model of the Virtual family head models [41] (age: 34, height: 1.74 m, weight: 70 kg, body mass index (BMI): 23.1 kg/m^2^). The 22 tissue volumes, identified by its constitutive properties—conductivity and dielectric constant (σ, ε_r_)—of the different head models are shown in Table 1. The head models were created by morphing the base model (H2) to produce models (H1-5) that varied in shape/eccentricity and volume and weights.

**Table 1:** Anatomically detailed head models. The 22 tissue volumes (inside each head model), identified by their constitutive properties (conductivity and dielectric constants) (σ, ε_r_) of the different head models H1-5, are shown. The anatomically detailed head models H1-5 were created by morphing the Duke male model (H2) of the Virtual family head models (34). Models are arranged in increasing volume/mass.

Epsilon	Sigma	H1	H2	H3	H4	H5
5.64	0.04	126.5	131.6	143.0	141.4	160.9
5.76	0.03	514.6	503.9	475.0	493.2	529.4
13.45	0.08	561.6	582.2	617.5	612.6	690.1
26.82	0.29	169.8	174.3	189.6	186.3	211.2
36.95	0.42	444.8	472.3	507.5	492.7	566.1
43.82	0.41	17.7	15.0	10.8	13.2	12.1
46.81	0.55	33.0	26.9	18.5	21.7	21.1
48.00	0.54	30.7	24.6	19.2	22.2	21.1
48.97	0.65	29.3	29.9	32.6	31.2	36.4
49.90	0.64	42.6	40.6	35.6	36.6	40.4
51.96	0.55	404.1	404.1	401.7	404.7	443.4
51.96	0.63	0.2	0.2	0.3	0.3	0.3
58.23	0.77	529.2	547.6	588.1	580.5	659.1
58.93	0.74	110.0	114.6	124.4	118.9	139.3
58.93	0.97	998.8	974.2	905.1	954.0	1011.7
59.82	0.97	1.2	1.0	1.1	1.2	1.0
61.43	1.15	130.8	137.0	148.1	143.0	164.4
62.47	0.85	2.7	3.3	3.5	3.9	3.7
65.69	1.32	0.4	0.5	0.4	0.5	0.5
68.74	0.97	19.3	18.8	15.7	17.9	17.5
69.02	1.52	7.1	7.6	8.6	8.1	9.3
72.78	2.22	211.7	216.9	237.6	232.3	265.5
**Dielectric Constant, and Conductivity**		**4387**	**4427**	**4484**	**4516**	**5005**
		**Individual Tissue Volumes and Total Volume (Cubic Cm) for each Head Model**				

The maximum variation in the mass was 14% and changes in shape was quantified by eccentricity of the head. The eccentricity measure is defined as the ratio of major to minor axis at the eye brow level and was obtained from the cross-sectional area and perimeter estimate at the same location. This was achieved using the DIP library [42] where the method1 by Proffitt et.al. [43] used different weights for inclined boundary lines, and the method2 by Vossepoel et.al. [44] used corner correction to obtain the perimeter of odd shapes. Eccentricity of heads varied from circular (eccentricity ~ 1) to elliptical (eccentricity ~ 1.4). Fig 1(C) shows the contours of the different head models (axial, coronal and sagittal boundary of brains and heads) used in the FDTD simulations. The relative permittivity map is also shown in Fig 1(B).

The perimeter of the models and their eccentricity when compared with those measured on the 4 volunteers indicate the shape and volume of heads used in the RF simulations represent a comprehensive sample of an adult population who might undergo an MRI [45].

**Software.** FDTD models of the TTT and TEM head coils i.e. the coil geometry: including the coaxial transmission lines, RF shielding, anatomically detailed head models and the terminating (perfectly matched layers) PML [46] are shown in Fig 1(A). The 3-dimensional computational domains with isotropic resolution of ~1.6mm was constructed by setting the constitutive properties to that of the RF coil (comprising of Rexolite or, Teflon, or Copper) or to the different tissues of the anatomically detailed head models. The coil model has a true transmission line model for the excitation elements with accurate modeling capability of the coil’s input impedance and coupling [36, 47–49].

The numerical models of the coils were tuned to Larmor frequency of 7T (297 MHz) using head model (H2) by adjusting the gap between the inner coaxial elements of the TTT and TEM elements and the modeling of the excitation source(s) while observing the scattering (S) matrix of the true transmission line model. The same tuning configuration applied on H2 is utilized for all other head models without re-tuning/matching for each individual head model.

Note that the full wave 3D FDTD models of the TTT and the TEM coils and the generic framework of validating experiment with simulation studies of the transmit field, input impedance and coupling between coil elements have been performed and validated in earlier studies [36, 48, 49].

#### Excitation strategy

The fields (electric and B_1_^+^) associated with the individual ports of each coil are combined using:

1) quadrature (TEM) and pseudo-quadrature (TTT) excitation, and 2) RF shimming (optimized excitation using phase-only or amplitude-and-phase excitation) for both the TEM and TTT coils. The RF shimming aims at achieving the lowest combination of the coefficient of variation (CV) and maximum to minimum (max/min) inside the region of interest (ROI) for all 5 head models. The ROI is the volume encapsulating the whole head above and including the temporal lobes and cerebellum while excluding the ears and the nasal cavity. The minimization of the CV and max/min inside the ROI is achieved by constraining the mean transmit B_1_^+^ field intensity to 11.74 μT, which results in 180^o^ flip angle with 1 ms square pulse, using a ~ 4.4 kW RF amplifier capacity (45% power loss from a standard 8 kW RF amplifier to the coil ports). Note that the phase-only shim cases (quadrature and phase-only arrangements) can be readily implemented in the combined mode of the MRI system without the need for parallel transmission mode.

RF absorption in the whole head quantified by SAR (W/Kg) averaged over any 10g of tissue was obtained for a continuous wave with mean transmit B_1_^+^ field intensity of 2μT in the ROI.

### Experimental measurements

#### Network analyzer measurements

This study was approved by the University of Pittsburgh’s Institutional Review Board (IRB PRO17030036) and involved four volunteers with approved written consent. The individuals have given a written informed consent to publish the details in this manuscript. Bench measurements were performed using a calibrated network analyzer (E5062A and 87050E multiport test set; Agilent Technologies Inc., CA, USA) together with an 85032B S-parameter test set. The Smith Chart measurements of different excitation ports of the TTT and TEM coils were recorded for the four volunteers (without re-tuning/re-matching of the excitation ports between different heads). Thus, both measurements in-vivo on the four volunteers, and with the five head models (H1-5) in FDTD simulations, were obtained without re-tuning/re-matching of the excitation ports.

#### In-vivo B_1_^+^ field measurements

To achieve the desired phase shifts, the quadrature (for TEM) and pseudo-quadrature (for TTT) cases were implemented by adjusting the lengths of the coaxial cables feeding the coils. Imaging was performed on two volunteers using a 7T scanner (Siemens Medical Solutions, Germany). B_1_^+^ maps were acquired with a turbo FLASH sequence with the following parameters: number of flip angles = 6, TR = 2.2 sec, TE = 1.4 msec, FOV = 220 mm, Matrix = 64x64, slice thickness = 3.2 mm and bandwidth = 510 Hz/pixel. The images from the six measurements of the B_1_^+^ map sequence was summed and used to create a brain mask. FSL’s brain extraction tool (BET) [50] was used to create a brain mask (bet -m -f 0.4 options were used). The created brain masks were visually inspected and corrected manually using ITK SNAP tool [51] in the regions where automatic segmentation of the brain failed.

## Results

### Input impedance of the loaded coils

**Fig 2:**
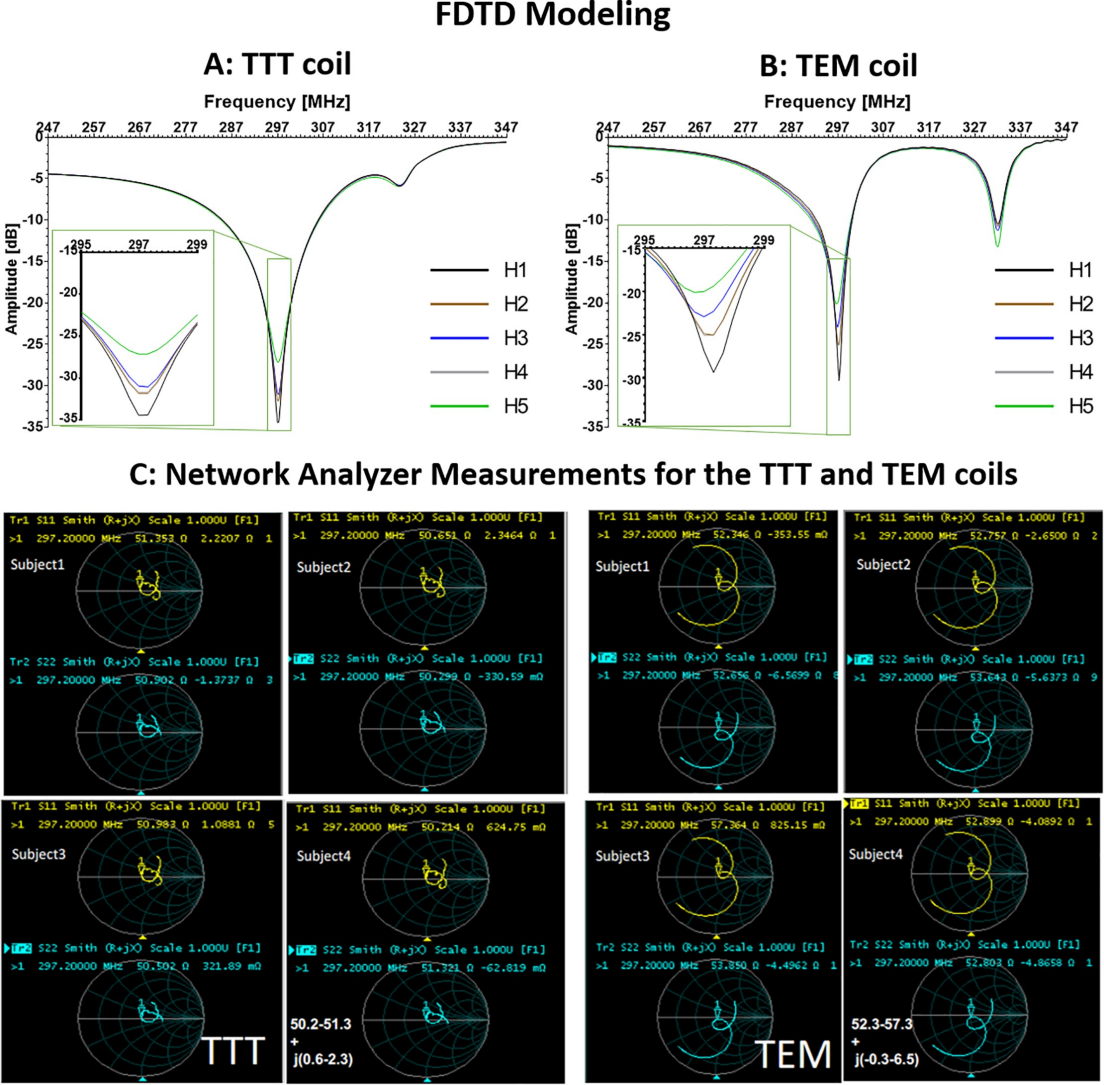
Simulations and network analyzer measurements. In A), reflection (Sxx) of a representative port of the TTT coil with the 5 different head models (H1-5) using full wave FDTD simulations. The maximum variation (between different head models) in input impedance amongst all the ports using the five different head models (80 cases) was 2.3%; In **B),** reflection of a representative port of the TEM coil with the 5 different head models using FDTD simulations. The maximum variation (between different head models) in input impedance amongst all the ports using the five different head models (20 cases) is 3.5%. There was no re-tuning or re-matching for any of the ports in both coils; the coils were tuned/matched to H2 and used in the same configuration for the other head models; In **C),** experimental impedance measurements (Smith Chart) for 2 representative ports for the TTT & TEM coils. Both coils showed consistent (< 5% variation) input impedance among all four volunteers.

Fig 2(A) and 2(B) show the FDTD calculated reflection coefficients (Sxx) and input impedances of different excitation ports in the TEM and TTT coils and the input impedances (real ± imaginary Ω) with the five head models (H1-H5). The displayed two (for the TEM coil) and four (for the TTT coil) reflection spectrums are representative of all the excitation channels of the TEM and TTT coils. Fig 2(C) also shows the bench measurements (using network analyzer) of the input impedances (Smith Chart) associated with two representative channels of the TEM and TTT coils on four different volunteers.

The numerical (across five different head models) and experimental (across four volunteers) results show that the reflection coefficient (Sxx) and input impedance values of both the TTT and TEM coils did not change appreciably. In terms of the input impedance, the maximum variation (between the five different head models) was 3.5% for the TEM coil (twenty cases represented by four excitation channels and five different head models) and 2.3% for TTT coil (eighty cases represented by sixteen excitation channels and five different head models.) The maximum variation measured with the network analyzer was 5%.

### Numerical and In-vivo B_1_^+^ field distributions and intensities

**Fig 3:**
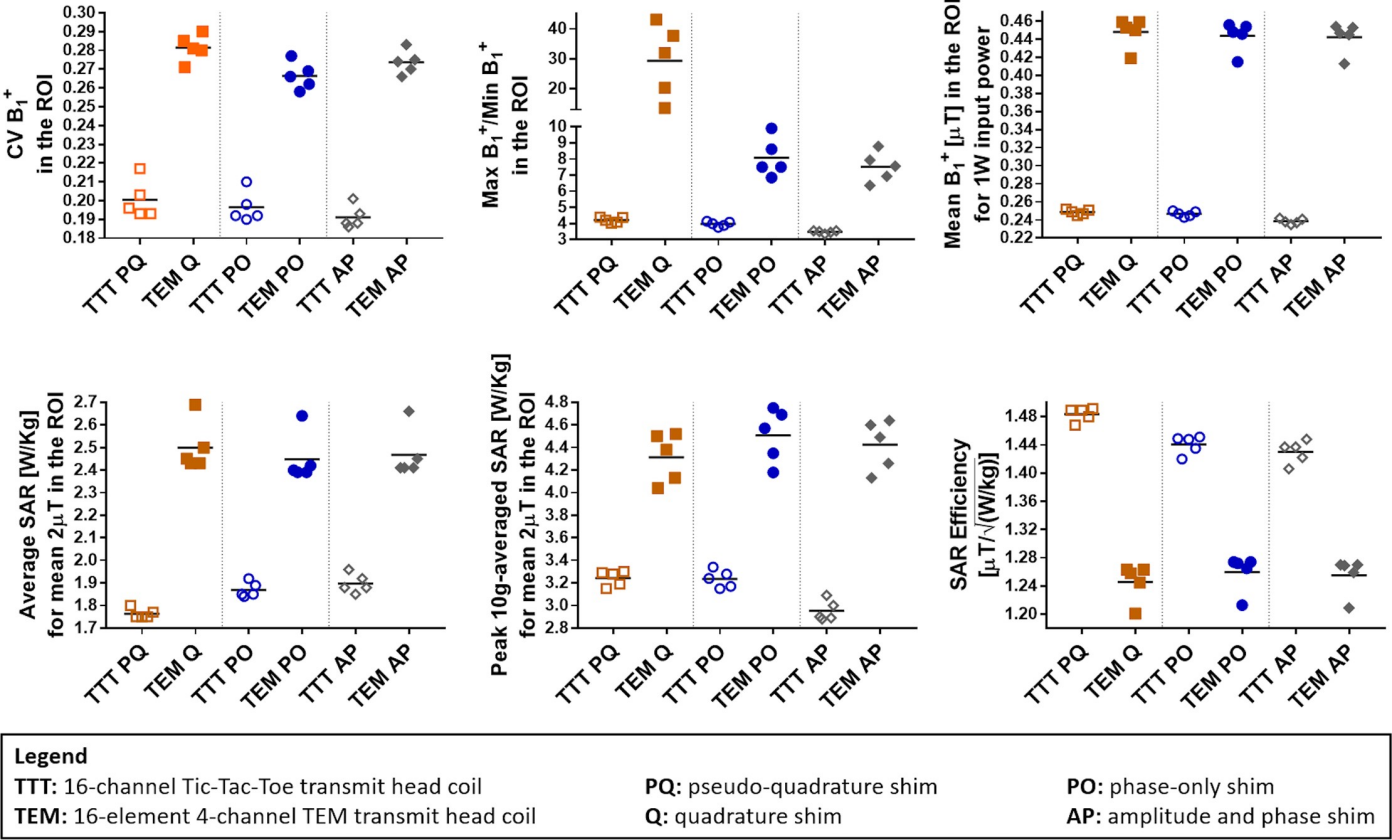
Performance comparison of the TTT and TEM coils with three different RF shimming techniques: Quadrature/pseudo-quadrature excitation, phase-only RF shimming (per coil, one RF shim set is applied to all 5 head models), and amplitude-and-phase RF shimming (per coil, one RF shim set is applied to all 5 head models). FDTD Calculated stats for the B_1_^+^ field and SAR for the five head models described in Fig 1 and Table 2 are shown. B_1_^+^ field homogeneity is quantified in terms of max/min, and CV in the region of interest (ROI). The ROI is defined as the whole head above and including the cerebellum and excluding the nasal cavities for all head models. The SAR performance is presented in terms of relationships between peak local SAR, average SAR, and B_1_^+^ field. Each line in each subfigure represents the mean value.

**Fig 4:**
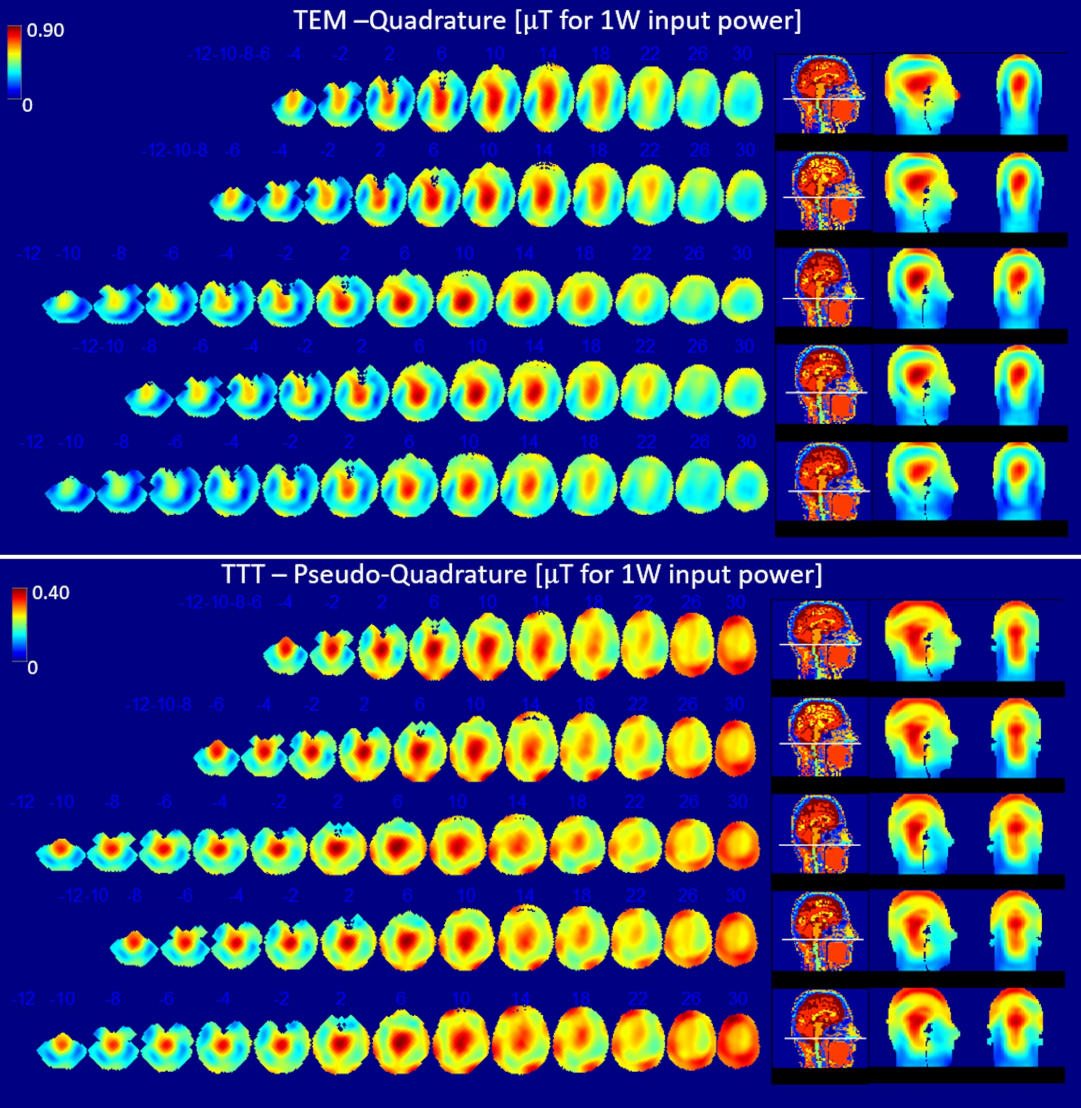
B_1_^+^ distribution for quadrature (TEM) and pseudo-quadrature (TTT) excitation. FDTD simulation data matching stats and conditions described in Table 2 and Fig 3. Axial slices were plotted with steps of 6.35 or 12.7 mm, slice numbers are indicated on top of each slice for instance (30, 26, … 2, -2, -4, …-10 etc.). Slices are plotted every 6.35 mm from (slice -10) through (slice 2) to capture the end of cerebellum in the head model, and every 12.7 mm subsequently to visualize the B_1_^+^ field distribution for the five head models.

**Fig 5:**
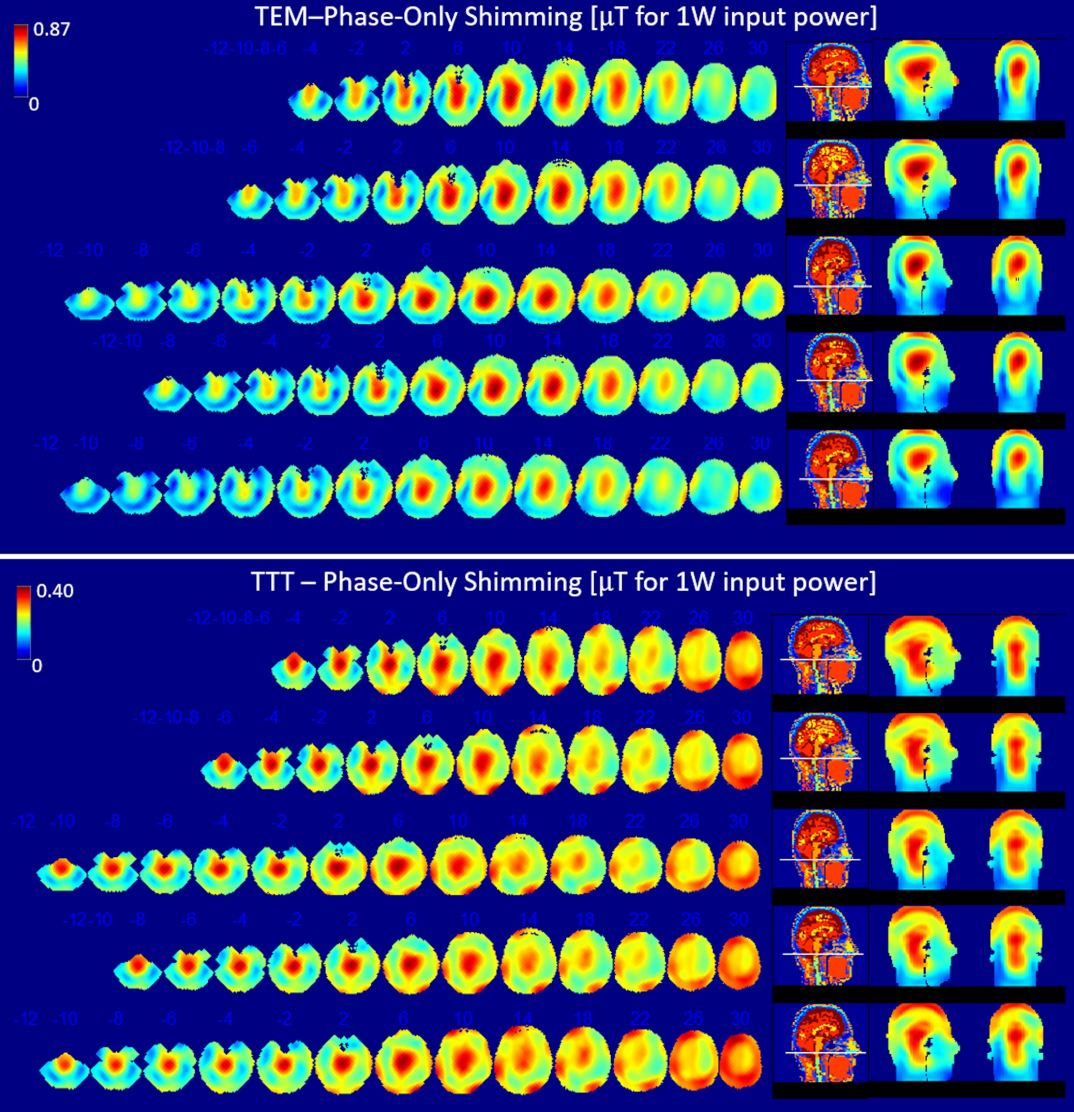
B_1_^+^ distribution for phase-only RF shimming. FDTD simulation data matching stats and conditions described in Table 2 and Fig 3. Description is provided in Fig 4 caption.

**Fig 6:**
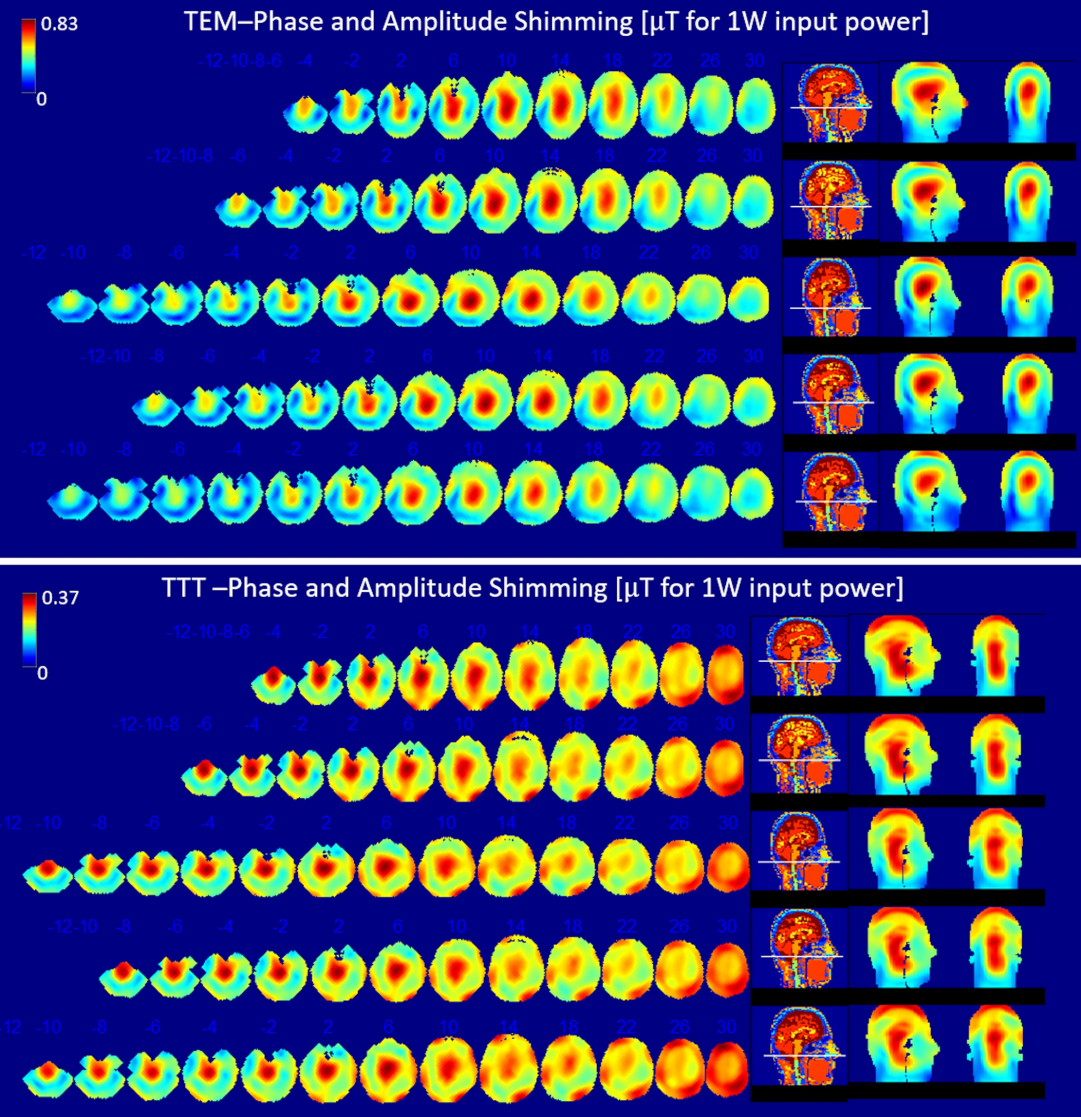
B_1_^+^ distribution for amplitude-and-phase RF shimming. FDTD simulation data matching stats and conditions described in Table 2 and Fig 3. Description is provided in Fig 4 caption.

Transmit B_1_^+^ field distributions across the five head models are shown for different excitation strategies (Figs 3–6 and Table 2). We compared three different excitation sets including quadrature, and variable amplitude-and-phase, and phase-only cases for the two coils. Due to the consistent tuning, and matching for both coils across different subjects, the coils were not re-tuned/matched in the simulations (five head models).

**Table 2:** Statistics for TEM and TTT coils for quadrature and pseudo-quadrature excitation, phase-only RF shimming, and phase-and-amplitude RF shimming. The average head model mass is 4.56kg (with 14% maximum variation among the models), the average brain volume is 1.51 L (with 15% maximum variation), and the average Eccentricity (major/minor axes) is 1.25 (with 37% maximum variation).

		B_1_^+^ Uniformity				Mean B_1_^+^ in the Same Volume (*μ*T) for 1 W Input Power		Peak SAR over Average SAR		Average SAR (W/Kg) for Mean B_1_^+^ = 2*μ*T in the Same Volume		Mean B_1_^+^ in the Same Volume over Sqrt(SAR) ($\mu T\sqrt{Kg}/\sqrt{W}$)	
		Maximum Intensity over Minimum Intensity in Whole Head above & Including Cerebellum and Excluding Nasal Cavity (Max B_1_^+^/Min B_1_^+^)		Standard Deviation over Mean in the Same Volume (CV)									
		TTT	TEM	TTT	TEM	TTT	TEM	TTT	TEM	TTT	TEM	TTT	TEM
**Quadrature (TEM) and Pseudo-Quadrature (TTT)**	**Avg.**	4.21	29.32	0.201	0.281	0.249	0.448	3.24	4.31	1.76	2.50	1.483	1.246
	**Max Variation (%)**	9.28	216.99	12.44	6.98	3.01	9.40	4.94	12.05	3.15	10.54	1.56	5.14
**Phase-only RF Shimming**	**Avg.**	3.97	7.94	0.197	0.266	0.247	0.451	3.24	4.58	1.87	2.40	1.441	1.271
	**Max Variation (%)**	10.29	44.54	10.47	7.46	2.99	9.83	6.26	13.60	4.48	10.37	2.21	5.06
**Phase-and-Amplitude RF Shimming**	**Avg.**	3.47	7.51	0.193	0.273	0.240	0.443	2.95	4.42	1.90	2.47	1.430	1.267
	**Max Variation (%)**	6.13	38.2	8.22	6.26	2.90	9.89	7.5	12.5	6.06	10.37	2.98	5.06

#### B_1_^+^ field homogeneity

Homogeneity of the B_1_^+^ field distribution was calculated in terms of maximum over minimum B_1_^+^ (max/min) and coefficient of variation (CV) which is defined as the standard deviation over mean (**σ**/**μ**) in the ROI. Figs 4–6 show multiple axial, one coronal, and one sagittal slices of B_1_^+^ field distribution. Sagittal conductivity maps of the different head models are also plotted with a line indicating the end of cerebellum in each of the five head models. The ROI, where stats were obtained in the five head models (Fig 3 and Table 2), includes the whole head above and including the cerebellum and excluding the nasal cavities and ears. The ROI volume of the different head models are different as indicated by the different axial slice number # which determines the end of cerebellum for each head model.

Fig 3 and Table 2 provides details of the following stats. In terms of quadrature (TEM) and pseudo-quadrature (TTT) excitation, the average (across H1-5) CV and max/min values for the B_1_^+^ field distribution are 20/28% and 4.21/29.32 respectively for the TTT/TEM Coils. In terms of phase-only RF shimming, the average (across H1-5) CV and max/min values for the B_1_^+^ field distribution are 20/27% and 3.97/7.94 respectively for the TTT/TEM Coils. In terms of amplitude-and-phase RF shimming, the average (across H1-5) CV and max/min values for the B_1_^+^ field distribution are 19/27% and 3.47/7.51 respectively for the TTT/TEM coils.

In terms of CV, the TTT design provides 40%/35%/41% average (across H1-H5) percentage improvement over the TEM design for quadrature/phase-only/amplitude-and-phase- excitation strategies. In terms of max/min, the TTT design provides 597%/99%/116% average (across H1-H5) percentage improvement over the TEM design for quadrature/phase-only/amplitude-and-phase excitation strategies.

#### B_1_^+^ field vs. input power and B_1_^+^ field vs. SAR

We evaluated B_1_^+^ field efficiency based on SAR as well as input power. Table 2 provides relevant statistical details. In terms of quadrature (TEM) and pseudo-quadrature (TTT) excitation, the average (across H1-5) mean B_1_^+^ field intensity for 1 W input power and mean B_1_^+^ field intensity (in ROI) for 1 W average SAR ($\mu T\sqrt{Kg}/\sqrt{W}$)) (in whole head volume) are 0.25/0.45 [$\mu T/\sqrt{W}$] and 1.48/1.25 [$\mu T\sqrt{Kg}/\sqrt{W}$], respectively for the TTT/TEM Coils. Therefore, the TTT design has ~44% lower average B_1_^+^ intensity for 1W input power and ~18% higher average B_1_^+^ for 1 W/kg average SAR for this case. In terms of phase-only RF shimming, the average (across H1-5) mean B_1_^+^ field intensity for 1 W input power and mean B_1_^+^ field intensity for 1 W average SAR are 0.25/0.45 [$\mu T/\sqrt{W}$] and 1.44/1.27 [$\mu T\sqrt{Kg}/\sqrt{W}$], respectively for the TTT/TEM coils. Thus, the TTT design presents ~44% lower B_1_^+^ for 1W input power and ~13% higher B_1_^+^ for 1 W/kg average SAR for the phase-only cases. In terms of amplitude and phase RF shimming, the average (across H1-5) mean B_1_^+^ field intensity for 1 W input power and mean B_1_^+^ field intensity for 1 W average SAR are 0.24/0.44 [$\mu T/\sqrt{W}$] and 1.43/1.27 [$\mu T\sqrt{Kg}/\sqrt{W}$] respectively for the TTT/TEM coils. Therefore, the TTT presents ~45% lower B_1_^+^ for 1W input power and ~13% higher B_1_^+^ for 1 W/kg average SAR for the phase-and-amplitude RF shimming.

#### In-vivo B_1_^+^ field measurements

**Fig 7:**
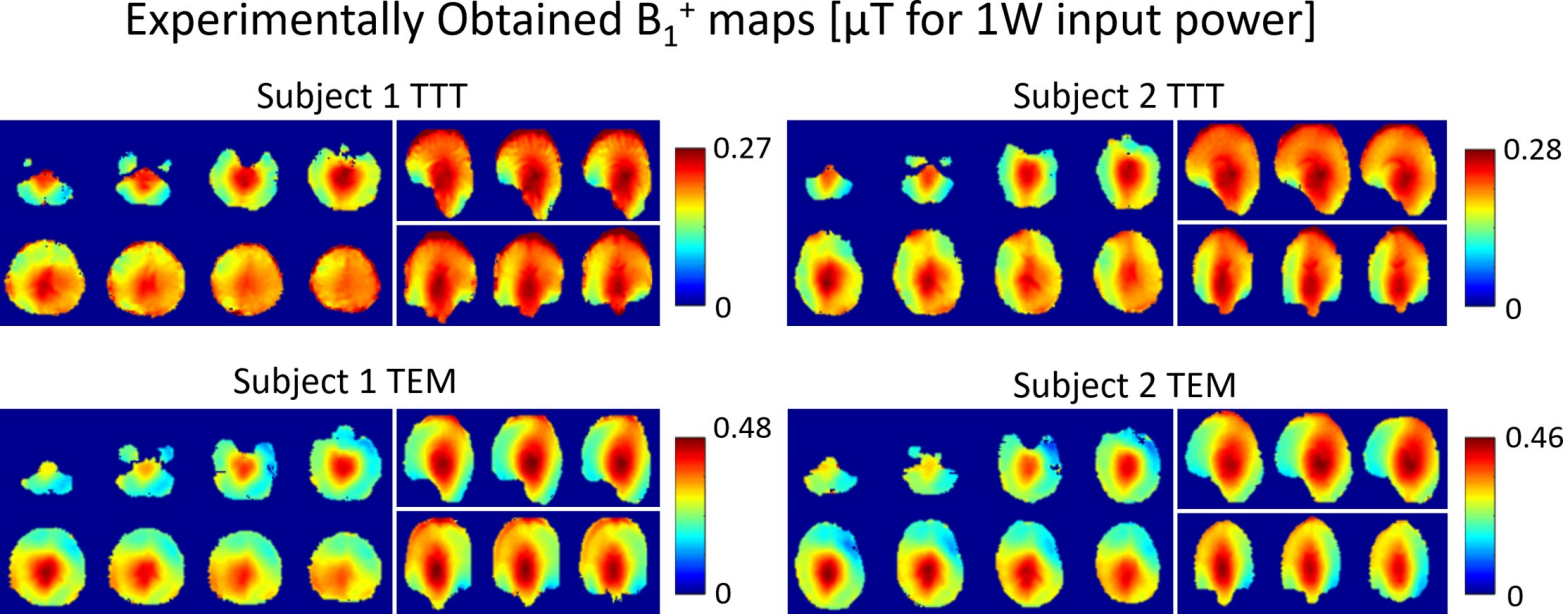
Experimentally obtained B_1_^+^ maps in two volunteers using the TEM and TTT coils. The color scale ranges from 0 to the maximum B_1_^+^ for each subject.

Consistent with the RF simulations, in-vivo measurements of transmit B_1_^+^ field distribution are shown in Fig 7 for the quadrature (TEM) and high flip angle pseudo-quadrature (TTT) excitation cases. The calculated CV for the TTT/TEM coils are ~21%/26% and ~20%/25% respectively for the two subjects (note that the utilized phases/cable lengths were the same for both subjects). Lack of sufficient B_1_^+^ field intensity in parts of the temporal lobe and cerebellum by the TEM coil makes measurement and use of max/min criterion inaccurate, and therefore it was not measured.

### Numerical SAR distributions and intensities

**Fig 8:**
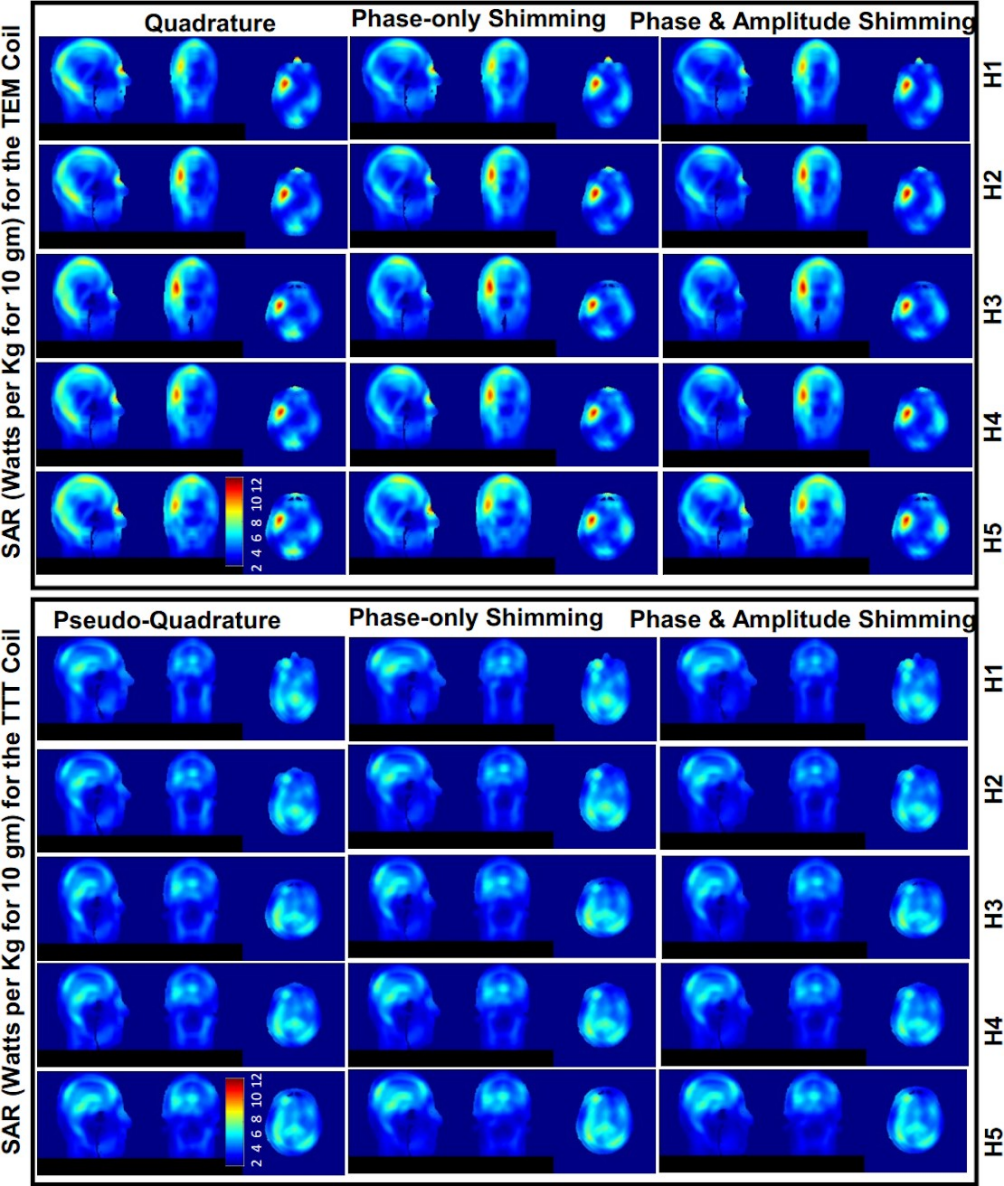
SAR (W/Kg for 10 g) distribution for 2*μ*T mean B_1_^+^ in the ROI in all head models H1-5 in the TEM and TTT coils. Exemplar axial, sagittal, coronal slices of SAR are shown. The distributions are plotted to the same maximum of 12 W/Kg for 10 g. The SAR is plotted for pseudo-quadrature arrangement, phase-only RF shimming (one RF shim set for coil applied on all 5 head models), and amplitude-and-phase RF shimming (one RF shim set for coil applied on all 5 head models). Please see Figs 2–6 and Table 2 for the conditions under which the SAR distributions are plotted.

SAR distributions across the five head models are shown for the three different excitation strategies (Fig 8).

#### Average SAR

We evaluated SAR efficiency based on calculating average SAR (across the whole head volume) for 2*μ*T mean B_1_^+^ field intensity in ROI. Table 2 and Fig 3 provide details of the following stats. In terms of quadrature (TEM) and pseudo-quadrature (TTT) excitation, the mean (across H1-5) average-SAR values for 2*μ*T mean B_1_^+^ field intensity in ROI are 1.76/2.5 W/Kg for the TTT/TEM coils. In terms of phase-only RF shimming, the mean (across H1-5) average-SAR values for 2*μ*T mean B_1_^+^ field intensity in ROI are 1.87/2.4 W/Kg respectively for the TTT/TEM coils. In terms of amplitude-and-phase RF shimming, the mean (across H1-5) average-SAR values for 2*μ*T mean B_1_^+^ field intensity in ROI are 1.9/2.47 W/Kg respectively for the TTT/TEM coils.

In terms of average-SAR values for continuous 2*μ*T mean B_1_^+^ field intensity in ROI, the TTT design provides 30%/22%/23% lower average (across H1-H5) values when compared to the TEM design for quadrature/phase-only/amplitude-and-phase excitation strategies.

#### Local SAR

We evaluated SAR distribution based on calculating local SAR (W/Kg for 10 g) to average SAR (W/Kg for 10 g) ratio. Table 2 and Fig 3 provide details of the following stats. In terms of quadrature (TEM) and pseudo-quadrature (TTT) excitation, the mean (across H1-5) local to average SAR ratio are 3.2/4.3 respectively for the TTT/TEM coils. In terms of phase-only RF shimming, the mean (across H1-5) local to average SAR ratio are 3.2/4.6 respectively for the TTT/TEM Coils. In terms of amplitude-and-phase RF shimming, the mean (across H1-5 local to average SAR ratio are 2.9/4.4 respectively for the TTT/TEM Coils. In terms of local SAR to average SAR ratio, the TTT design provides 25%/30%/34% lower average (across H1-H5) values when compared to the TEM design for quadrature/phase-only/amplitude-and-phase excitation strategies.

### Consistency of the RF field distributions

#### B_1_^+^ field

We evaluated the consistency of B_1_^+^ field based on the four-abovementioned criteria namely: CV, max/min, mean B_1_^+^ field intensity for 1 W input power and mean B_1_^+^ field intensity for 1 W average SAR. B_1_^+^ field consistency was based on calculating the maximum variation change among the five head models, i.e. for any criteria, maximum variation is defined as [maximum value–minimum value] over [minimum value]. In terms of CV, the maximum variation change is 12/7%, 10/7.5%, and 8.2/6.3% respectively for the TTT/TEM coils and for quadrature, phase-only, and amplitude-and-phase excitation strategies, respectively. For max/min, the maximum variation change is 9.3/217%, 10/44%, and 6.1/38% respectively for the TTT/TEM coils and for the three excitation strategies. For mean B_1_^+^ field intensity for 1 W input power, the maximum variation change is 3/9.4%, 3/9.8%, and 2.9/9.9% respectively for the TTT/TEM coils and for the three excitation strategies. For mean B_1_^+^ field intensity for 1 W average SAR, the maximum variation change is 1.6/5.1%, 2.2/5%, and 3.0/5% respectively for the TTT/TEM coils and for the three excitation strategies.

#### SAR

We evaluated the consistency of SAR based on the two-abovementioned criteria namely average SAR (in whole head volume) for 2*μ*T mean B_1_^+^ field intensity in ROI and local to average SAR ration. In terms of SAR (in whole head volume) for 2*μ*T mean B_1_^+^ field intensity in ROI, the maximum variation change is 3.1/10%, 4.5/10%, and 4.5/10% respectively for the TTT/TEM coils for quadrature, phase-only, and phase-and-amplitude excitation strategies. For local to average SAR ratio, the maximum variation change is 4.9/12%, 6.3/14%, and 6/14% respectively for the TTT/TEM coils for the three excitation strategies.

## Discussion

### B_1_^+^ field distribution

The overall results show that transmit B_1_^+^ field homogeneity measured by CV as well as max/min (maximum B_1_^+^ over minimum B_1_^+^ in the ROI) are substantially improved with the TTT coil when compared to the TEM coil. The TTT coil is well suited for exciting cerebellum and brain stem [32] at 7T. That being said, the TEM coil design with its arrangement of multiple transmission lines aims at providing increased excitation in the middle of the brain [31] as opposed to extended coverage in the peripheries.

Based on our simulations, the central positioning in conjunction with the same back-of-head positioning in XY plane for different head models within the coils is optimal for in-vivo MRI acquisitions for the TEM coil. Prior simulations [52] showed that a slightly shifted position in the XY plane provides moderate better coil performance for the TTT coil. To keep consistency between both coils, this shifted case (with the TTT coil) was not considered in this study.

### B_1_^+^ field vs. input power

The load insensitive nature of the TTT coil comes from having strongly coupled elements [53], which decreases the load to coil interactions. Although this comes at a price as its transmit efficiency evaluated as B_1_^+^/√forward power is substantially lower when compared to the TEM coil. That being said, the implementation of the TTT coil has been shown to provide adequate B_1_^+^ for inversion (180^o^ flip angle at 1 ms pulse width), and turbo spin echo and other clinical scans requiring inversion with a standard 8 kW RF amplifier [52, 54–56].

### SAR distribution and intensity

Despite lower ratio of B_1_^+^ field vs. input power for the TTT design, it provides ~22%-30% lower average (across H1-H5) SAR values (for 2*μ*T mean B_1_^+^ field intensity in ROI) when compared to the TEM design for quadrature and RF shimming excitation strategies. In terms of local SAR to average SAR ratio which can be the limiting factor in determining the allowed amount of tissue absorption, the TTT design also provides ~25%-34% lower average (across H1-H5) ratios when compared to the TEM for quadrature and RF shimming excitation strategies.

### Consistency of the B_1_^+^ field and SAR distributions and intensities

Strategies to overcome patient specific electromagnetic interactions at UHF include; building systems/sequences that are invariant across subjects and using a safety factor in SAR monitoring [57]. B_1_^+^ inhomogeneity can potentially be alleviated using a combination of multi-port transmission and/or by adiabatic/hyperbolic or composite pulse sequences that produce constant flip angle independent of transmit field [58]. However, to achieve optimal performance (image quality as well as safe RF levels), one must know how the RF fields produced by coils/arrays behave in every imaged subject prior to an MR experiment. The process of measuring and/or simulating the RF fields and implementing a targeted excitation profile is time consuming and often cannot be performed in real-time with the subject in the scanner. Additionally, the measurements may be inaccurate when signal voids exist in the images and cannot be generalized across subjects when the RF field produced by coil/array is widely varying across different subjects. Therefore, the coil performance across different subjects presented in this study becomes relevant for effective and safe coil use at UHF MRI.

This work shows that both the TEM and TTT coils showed consistent tuning and matching across different subjects/head models. Across 5 head models with brain volumes changing between 1330 and 1740 cc and eccentricity changing between 1.04 and 1.48. That being said, the variation in transmit B_1_^+^ field and SAR distributions/intensities (across different head models) for the TTT coil was substantially lower than the TEM coil.

Hot-spot changes are predicted to changes in boundary conditions due to significant changes in constitutive properties (σ, ε) and varying resistance to induced currents, in the different heads. These should result in different local SAR for the different heads. Thus, SAR accumulation or hot-spots at eye/nose sinuses, bone-CSF interface, among other regions, are anticipated for different head/(model)s as the boundary regions differ for each of them. For local to average SAR ratio, the maximum variation change is ~5.7%/13% respectively for the TTT/TEM coils.

## Conclusion

We studied the B_1_^+^ field produced in the ROI that encapsulates the whole head above and including the temporal lobes and cerebellum and excludes the nasal cavity, and SAR in the whole head volume. Statistics of the simulated B_1_^+^ field (efficiency, homogeneity, and consistency), and SAR (intensity and consistency of average and local values) across the 5 head models are tabulated in Table 2, shown in Fig 3, and further elaborated in the Results Section.

A direct comparison between the TTT and TEM coils for the phase-only shim case (directly implementable in the single transmit mode of the 7T scanner) shows the following observations.

1) Both coils present negligible variance of input impedance among different head models/subjects (<5%) in the simulated/measured data.

2) The TTT design shows substantial improvement in the B_1_^+^ homogeneity and consistency demonstrated in the max/min values of the TTT design (mean max/min = 3.97, maximum variation = 10% between different heads) when compared with that achieved with the TEM design (mean max/min = 7.94, maximum variation = 44%), which represents a significant a) excitation drop in the cerebellum and temporal lobes and b) variation between subjects.

3) The TTT design presents substantial improvement in the B_1_^+^ homogeneity, as demonstrated in the values of the CV (TTT = 0.20 mean, 10% maximum variation between different heads; TEM = 0.27 mean, 7.5% maximum variation). This is critical for achieving fidelity of the B_1_^+^ field distribution across the human head. The TTT design has a significant lower B_1_^+^/power efficiency, with mean B_1_^+^ = 0.25μT for 1W input power (with 3.0% maximum variation) against 0.45μT (with 10% maximum variation) for the TEM design; however, both coils present enough B_1_^+^ intensity to have spin inversion with 1ms square pulse and 8kW RF power amplifier including power losses. This could be critical when high peak B_1_^+^ intensity is needed with limited RF power amplifier capacity.

4) The TTT design has better SAR efficiency, with 1.44 μT/√(W/kg) (maximum variation = 2.2% between different heads) against 1.27 μT/√(W/kg) (maximum variation = 5.1%) for the TEM design. This is critical for high SAR acquisitions (turbo spin echo, FLAIR, DTI, etc …).

5) The TTT design has lower peak over average SAR ratio (3.24) with maximum variation = ~6.3% between different heads against 4.58 (which is ~41% higher than the TTT value) and maximum variation = ~14% for the TEM design. This is critical for high SAR acquisitions as well as meeting the regulatory limits for local and average SAR. Note that with the FDA-approved single transmit mode 7T systems, the scanner’s online SAR calculations do not significantly change between different human heads.

For the TTT coil, the lack of significant variation in terms of B_1_^+^ distribution/intensity and local/average SAR in different subjects translates to ease in set up (no B_1_^+^ maps and RF shimming for every subject are necessary). This is very important in a clinical environment when the time to scan a subject is very limited and any retuning or extra acquisition is costly. Moreover, the subject-insensitivity RF performance associated with the TTT design provides greater RF safety assurance measured by consistency in the local and average SAR with different subjects. With narrow tolerance parameters associated with TTT coil, the results also show that numerical simulations can be potentially performed on a representative head model without the need for 1) subject-specific transmit B_1_^+^ maps measured in-vivo or 2) subject-specific SAR calculations. Finally, the TTT and TEM RF coils can be used in either parallel transmit or in single transmit systems.
